# Integrins in the Spotlight of Cancer

**DOI:** 10.3390/ijms17122037

**Published:** 2016-12-06

**Authors:** Daniela Bianconi, Matthias Unseld, Gerald W. Prager

**Affiliations:** Department of Internal Medicine I, Comprehensive Cancer Center Vienna, Medical University of Vienna, A-1090 Vienna, Austria; daniela.bianconi@meduniwien.ac.at (D.B.); matthias.unseld@meduniwien.ac.at (M.U.)

**Keywords:** integrins, cancer, proliferation, invasion, apoptosis, telomerase, angiogenesis, apoptosis, contact inhibition, locomotion

## Abstract

Integrins are heterodimeric cell surface receptors that bind to different extracellular ligands depending on their composition and regulate all processes which enable multicellular life. In cancer, integrins trigger and play key roles in all the features that were once described as the *Hallmarks of Cancer*. In this review, we will discuss the contribution of integrins to these hallmarks, including uncontrolled and limitless proliferation, invasion of tumor cells, promotion of tumor angiogenesis and evasion of apoptosis and resistance to growth suppressors, by highlighting the latest findings. Further on, given the paramount role of integrins in cancer, we will present novel strategies for integrin inhibition that are starting to emerge, promising a hopeful future regarding cancer treatment.

## 1. Introduction

Integrins are obligate heterodimeric cell surface receptors, which are present in all nucleated cells of the human body. Each integrin consists of one of 18 α- and one of eight β-subunits, giving rise to a repertoire of 24 different integrins in mammals [1]. Integrins function as bridges between the extracellular matrix (ECM) and the cytoskeleton and work as radars that detect changes in the environment, enabling cells to react according the external milieu. Moreover, intracellular signaling or changes in the cytoskeleton can regulate the affinity of integrins to components of the ECM [2], enabling integrins with the ability to signal in both directions: in an outside-in and in an inside-out manner.

Integrins are involved in key developmental processes such as cell differentiation, cell adhesion, cell migration, cell proliferation and cell survival and are expressed in all metazoans. The diversity and at some extent, promiscuity, of the mammalian integrin subunits arose during evolution as organism complexity increased from genes that were already present in protozoa such as in the apusozoan protest *Amastigomonas* sp. [3,4]. Each cell type exhibit a specific range of integrins and this repertoire changes according to the cellular or environmental input. In cancer, malignant cells change this repertoire in response to changes in the components or stiffness of the ECM, in response to growth factors or due to intracellular alterations such as activation of oncogenes.

Over the past years, considerable progress has been made in describing integrin signaling pathways and new interaction partners of integrins. The scientific literature regarding integrins is overwhelming in such a manner, that the term data smog coined by the journalist David Shenk almost 10 years ago would perfectly apply in this context. Therefore, in this review we will highlight the crucial contributions of integrins in The Hallmarks of Cancer, which were proposed for the first time in the seminal article of Hanahan and Weinberg in 2000 [5,6]. We believe that the broad acceptance and the influential power of this article resides in the fact that the authors could group common characteristics of all cancer types together and classify them in only six hallmarks. Therefore, we will pinpoint the role of integrins in the hallmarks of cancer by discussing the recent advances on cancerous integrins, providing the reader with a clear and updated overview of the role of integrins in carcinogenesis. It is beyond the scope of this article to discuss meticulously integrin-mediated pathways and thus, we will summarize the principal signaling pathways to facilitate the reading of this review. Readers are referred to several articles that describe these mechanisms in detail.

### Giving Light to Life

When trying to understand the role of integrins, one should bear in mind that cells are per se sentenced to death. This means that cells need to receive inputs in order to live, proliferate, migrate and die in a controlled manner and that cells need integrin to sense these inputs; otherwise multicellular life would not be viable.

**Figure 1:**
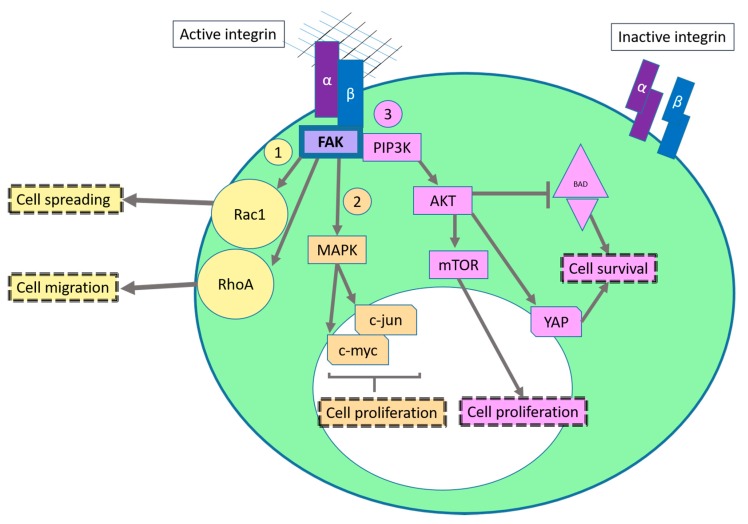
Schematic representation of the integrin outside-in signaling. Through the activation of focal adhesion kinase (FAK) via integrins, Src is activated (not shown). (1) Rac1 GTPase is recruited to the plasma membrane, GDP-GTP exchange occurs and controls actin assembly in nascent protrusions [11,27]. At later stages, RhoA activity increases, leading to the formation actin stress fibers and stimulates actomyosin contractility via its downstream effector Rho-associated protein kinase (ROCK) [28]; (2) Integrin mediated FAK activation triggers the mitogen-activated protein kinase (MAPK) pathway. Different transcription factors are phosphorylated, leading to the expression of pro-proliferation genes; (3) The PIP3K/AKT pathway activation leads to enhanced translation of pro-survival and pro-proliferation genes via the mammalian target of rapamycin (mTOR) pathway. The phosphatidylinositol-3-phosphate kinase/AKR mouse thymoma kinase (PIP3K/AKT) pathway cross-talk with the Hippo pathway via Yes-associated protein (YAP). YAP is a transcription factor that can induce for example expression of the anti-apoptotic proteins survivin and Bcl-xL [29]. (Of note, there are a plethora of cross-talks between all these pathways that are not discussed here for simplication purposes). Arrows: interaction with another protein or promotion of a specific cell behavior; T-bar: inhibition; dotted boxes: effect/consequence from the signaling cascade; big ellipse in green: cell; small ellipse in white: nucleus.

The most studied integrin mediated pathway is probably the focal adhesion kinase (FAK) signaling pathway (Figure 1). Upon binding to its specific ligand, integrins cluster together and the cytoplasmic tail of the β-subunit interacts physically with the four-point-one, ezrin, radixin, moesin (FERM) domain of FAK, displacing it and allowing autophosphorylation of the tyrosine residue 397, which act as docking site for members of Src family of tyrosine kinases that phosphorylate further tyrosine residues (Y576 and Y577) leading to maximal FAK activation [7,8,9]. All adherent cells exhibit increased activation of FAK [8]. The FAK-Src complex has multiple downstream effectors as summarized below.

Activated FAK-Src complex promotes the activity of a GTPase which belongs to the Ras superfamily of small GTP-binding protein known as Rac1 (Ras-related C3 botulinum toxin substrate 1) that stimulates protrusion formation by stimulating actin polymerization [10,11]. Rac1 activation is involved in spreading and in the early stages of migration. At the same time, Src can suppress the activity of the RhoA GTPase upon binding to fibronectin via α5β1 [12]. This relieves cytoskeletal tension, allows cell spreading and inhibits migration [12,13]. At later stages of cell spreading or for instance, by constitutive activation of αvβ3 via ligand binding, RhoA activity leads to the formation of stress fibers and promotes migration (Figure 1(1)) [12,14]. The reader can find an excellent explanation of this interplay in [15] and a detailed description of integrin mediated cell migration here [16].

In addition, phosphorylation of FAK leads to the Ras-mediated activation of the mitogen-activated protein (MAP) kinase pathway (MAPK/ERK pathway), which is associated with proliferation and tumorigenic behavior (Figure 1(2)). Through this pathway several transcription factors such as the oncogene *c-myc* and *c-jun* are activated via phosphorylation and therefore, the activation of the MAPK pathway leads to the transcription of genes that are important for cell proliferation and cell cycle progression [17,18]. This pathway can be activated by cell adhesion (e.g., binding of α5β1 to fibronectin) or growth factors (such as epidermal growth factor (EGF)) [18].

Moreover, phosphorylated FAK acts as docking site for phosphatidylinositol 3-kinase (PI3K), which leads to the activation of the serine/threonine protein kinase AKT (AKR mouse thymoma kinase) via phosphoinositide-dependent kinase-1 (PDK1) [19,20]. In cancer, AKT signaling pathway (Figure 1(3)) can be activated through αvβ3 integrin and promotes for example survival by targeting the pro-apoptotic Bcl-2 related protein known as Bcl-2-antagonist of cell death (BAD) [21]. In addition, if cells have sufficient nutrients, AKT activation leads to the activation of the mammalian target of rapamycin (mTOR) pathway, which enhances mRNA translation of pro-proliferating, pro-migrating and pro-survival genes (such as vascular endothelial growth factor (VEGF)) and lipid synthesis [22,23]. The AKT signaling pathway can also lead to the phosphorylation of Yes-associated protein (YAP) which acts as an apoptotic suppressor [21]. The activation of YAP represents a cross-talk with a newer signaling pathway known as Hippo pathway. This pathway controls organ size by regulating cell proliferation and apoptosis [24]. It consists of more than 30 components which have two major downstream effectors: YAP and TAZ (transcriptional coactivator with PDZ-binding motif) [24,25]. When these transcriptional coactivators translocate into the nucleus, they bind to TEA domain (TEAD) transcription factor family and induce expression of genes that promote proliferation, survival and migration (such as c-myc and survivin) [24,26].

The cytoplasmic tails of β1 and β3 can also interact with the serine/threonine kinase ILK (integrin-linked kinase), which is activated by binding phosphatidylinositol-3-phosphate (PIP3) and leads to the activation of AKT, glycogen synthase kinase 3 (GSK3) and phosphatase holoenzyme inhibitor 1 (PHI-1) [30,31]. Under normal conditions, ILK signaling is involved in nerve growth factor-stimulated neurite outgrowth and as it was recently reported, in wound repair by regulating fibroblast migration and its differentiation to myofibroblasts [32,33].

**Figure 2:**
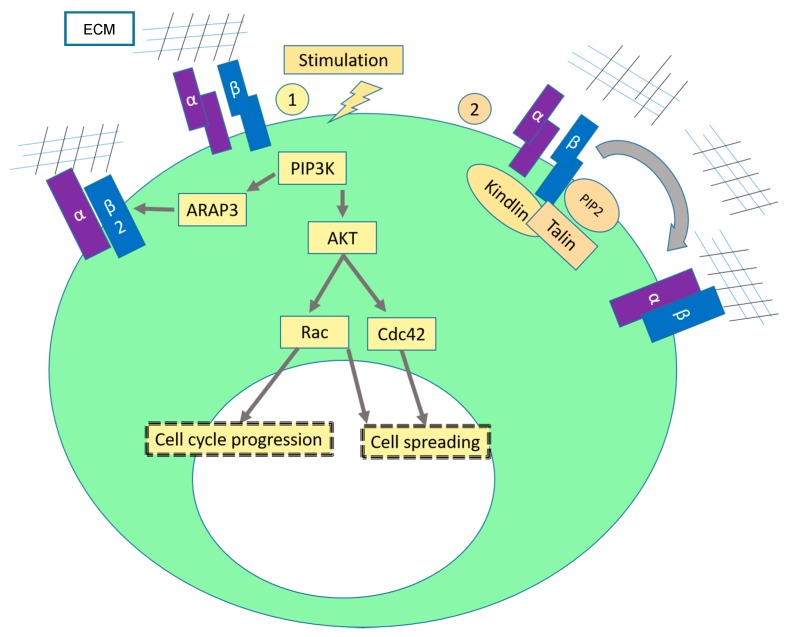
Inside-out Integrin mediated cell spreading. Cell spreading is described as the deformation of the plasma membrane due to extensions of protusions, leading to the adhesion between cell and substrate [44]. (1) Upon stimulation with cytokines, PI3K is activated. On the one side, PI3K activation phorphorylates AKT and the downstream effectors Rac and Cdc42 are activated [45]. These are small GTPases that are involved in the reorganization of the cytoskeleton which leads to the formation of lamelipodia and filipodia. On the other side, it was shown that PI3K induces activity of the β2 integrin subunit via ARAP3 (Arf GAP and Rho GAP with ankyrin repeat and PH domain 3) [46]. ARAP3 is Rap-regulated GTPase-activating protein for RhoA and Arf6 which are resposible for actin modulation [47,48,49]; (2) Accumulation of PIP2 leads to the recruitment of talin to the plasma membrane, enabling a physical interaction between talin and the β1 and β3 integrin subunits which induces a conformational change in the integrin heterodimers that increases integrin affinity to its ligand. Kindlins are also essential for the inside-out activation of integrins [50]. In fibroblasts, kindlin-2 binds and activates FAK, inducing formation of lamellipodia [51]. Arrows: interaction with another protein or promotion of a specific cell behavior; T-bar: inhibition; dotted boxes: effect/consequence from the signaling cascade; big ellipse in green: cell; small ellipse in white: nucleus.

Several lines of evidence suggest that cell spreading might occur primarily via the “inside-out” integrin signaling pathway as depicted in Figure 2. Unlike what happens in the outside-in pathway, integrins are not directly activated by extracellular ligands but by intracellular events, which activate or modulate integrin ligand affinity. In resting cells, integrins are present on the plasma membrane in a low-affinity state and when they are needed, they are activated (reviewed in [2,34,35]). Talin is the major intracellular activator of integrins [34]. Upon activation of talin, talin is recruited to domains of the plasma membrane rich in phosphatidylinositol 4,5-bisphosphate (PIP2) and pulls the cytoplasmic β tail of integrins inducing a conformational change that allows ligand binding (active form) (Figure 2(2)) [34]. Kindlins (kindlin-1, kindlin-2 and kindling-3) are other intracellular proteins of primordial importance in the modulation of integrin affinity [36]. These proteins are located at focal adhesion sites bound to the integrin cytoplasmic β tails and to the cytoskeleton and are essential for integrin signaling [37]. Loss of kindlins leads to integrin signaling dysregulation. For instance, it was reported that, knockout of kindlin-2 in mice leads to peri-implantation lethality caused by detachment of cell from the basement membrane [38]. A paper recently published, demonstrated that loss of kindlin-1 leads to the inhibition of keratinocyte electrotaxis (as it was also observed by inhibition of β1 integrins) [39]. In leukocytes, kindlin-3 activates integrin α4β1 of resting cells to adhere to vascular cell adhesion molecule-1 (VCAM-1) [40]. Studies in hemepatopoetic cells have previously shown that PI3K activity modulates the conformational changes of β1-integrins upon stimulation with pro-coagulation and pro-inflammatory factors [41,42,43] (Figure 2(1)). These observations were confirmed in adherent cells [43].

## 2. Hallmarking Cancer

The term cancer describes a variety of diseases that have one common feature: the loss of control. Tumor cells stop performing their specific functions and start an independent behavior regardless the signals they receive from the environment. Despite the infinite malignant cell behaviors and disease heterogeneity, Hanahan and Weinberg proposed for the first time in 2000 a model consisting of six essential acquired properties that are shared by all tumor cells [5]. These Hallmarks of Cancer are sustained proliferation, self-sufficiency, invasion and metastasis, limitless replicative potential, promotion of angiogenesis and evasion of apoptosis [5]. In this review, we will discuss how integrins contribute to all these hallmarks, playing a paramount role in tumorigenesis.

### 2.1. Sustaining Proliferative Signaling

Mature differentiated epithelial cells are quiescent and anchored in the ECM via integrins. Growth of postmitotic tissue resides mainly on growth of mass (hypertrophy) via the PI3K-Akt-mTOR pathway and not on an increase in cell number [52,53]. A strict control of cell proliferation is essential in multicellular organisms to ensure correct tissue function. In this context, integrins expressed in normal tissue play an essential role by mediating firm adhesion to the ECM ensuring tissue integrity.

**Figure 3:**
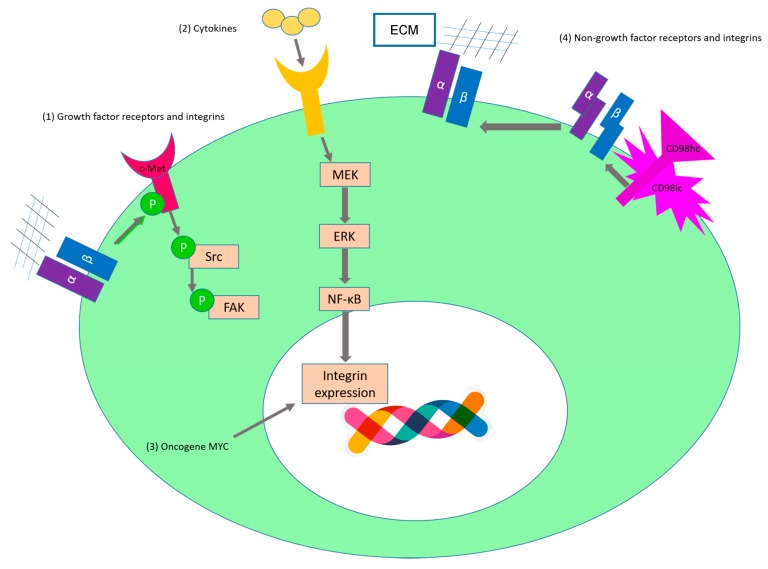
Role of integrins in tumor cell proliferation. (1) The crosstalk between integrins and growth factor receptors (summarized in Table 1) promotes cancer cell proliferation. For instance, in ovarian cancer cells c-Met is activated via phosphorylation upon binding of α5β1 to fibronectin [65]. This activation leads to activation of Src and FAK and promotes proliferation and invasion [65]; (2) Tumor necrosis factor α (TNF-α) leads to an increased expression of integrins such as α2β1 and αvβ3 [93,101]; (3) The oncogene MYC (v-myc avian myelocytomatosis viral oncogene homolog) can bind to promoter elements of α1β1 and leads to enhanced transcription of this integrin [87]; (4) Integrins can interact with transmembrane receptors such as CD98hc. CD98hc modulates integrin signaling by o conferring integrins with “stiffness” that allows them to trigger their signaling pathway [75]. (Of note, the DNA cartoon was designed by Freepik). Big ellipse in green: cell; small ellipse in white: nucleus.

In cancer, the strict control of proliferation is lost due to extrinsic factors such as the presence of mitogenic compounds (growth factors, cytokines or exogenous substances) or intrinsic factors such as activation of oncogenes, converting cancer cells in a self-sufficient entity. In this context, integrins play a crucial role by directly promoting proliferation or indirectly, by interacting with growth factor receptors [54]. The contribution of integrins to the promotion of cell proliferation is illustrated in Figure 3. The crosstalk between growth factors and integrins has been reviewed in detail in [55,56,57,58] and therefore we will not discuss exhaustively these interactions here. Table 1 highlights some interactions between growth factor receptors and integrins in cancer that are involved in proliferation. Three types of interactions can be distinguished: (1) direct interaction; (2) modulation of expression levels and (3) reciprocal activation. In the first type of interaction, there is a physical contact between a specific integrin and a growth factor receptor (sometimes via another protein or growth factor), which leads to the formation of a complex and a potentiation of the respective signaling pathway. The second type refers to an indirect interaction, by which integrins modulate the expression level of the growth factor receptor, for instance by modulating the turnover of the growth factor receptor. The third type implies that the activation of the growth factor receptor leads to integrin activation and vice versa.

The two most prominent integrins involved in tumorigenesis are αvβ3 and αvβ5. Integrin αvβ3 is expressed at low levels on resting epithelial cells, however it is overexpressed in a wide variety of cancers. In ovarian cancer, integrins αvβ1 and αvβ3 were shown to enhance proliferation via ILK and blocking αv was sufficient to arrest cell cycle [59]. Further on, evidence suggest that αvβ3 also regulates epidermal growth factor receptor (EGFR) promoter activity and influences co-clustering of the receptor on the ovarian cancer cell surface [60]. In vitro experiments have shown that normal human gland thyroid cells express αvβ3 upon treatment with hepatocyte growth factor/scatter factor (HGF/SF) [61]. In accordance with these findings, papillary thyroid carcinoma cells indeed express HGF and αvβ3, leading to an autocrine loop that controls integrin activation and enhancing proliferation [61]. Loss of salt bridge formation in the cytoplasmic tail of αvβ3 enhances adhesion to vitronectin, recruits talin and induces proliferation [62].

We would like to underline that integrins have also other interaction partners that are essential for integrin-mediated cell behavior (reviewed in [70,71,72]). One of them is the heterodimer amino acid transporter CD98, which consists of one light and one heavy chain [73]. While the light chain is responsible for the amino acid transport, the heavy chain (CD98hc) interacts with the cytoplasmic tails of the β1 and β3 integrin subunits [74] and confers integrins with “stiffness” that allows them to trigger their signaling pathway [75] (Figure 3(4)). Noteworthy, the interaction between CD98hc and integrins and subsequent activation of PI3K was found to be necessary for malignant transformation [76,77]. In line with these findings, we and others identify CD98hc as marker of different tumor types, such as pancreatic [78], prostata, gastric, tongue and renal cancer (see [79,80,81,82,83,84,85,86]).

It is well known that the activation of proto-oncogenes leads to carcinogenesis. To date, there is only one oncogene known that directly leads to an enhanced transcription of cancerous integrins. It was recently shown that the oncogene MYC (v-myc avian myelocytomatosis viral oncogene homolog) leads to an upregulation of α1β1 integrin by binding to two promoter elements [87] (Figure 3(3)). Integrin α1β1 can regulate collagen synthesis and might confer cells with the unique ability to adhere and migrate on collagen IV [88]. In addition, β1- and αv-ligand binding can induce activation of the EGF-receptor in the absence of ligands, leading to the activation of the MAP pathway and facilitating proliferation [89]. Noteworthy, α1β1 integrin negatively regulates EGFR under normal conditions. However, deletion of α1 in mesangial cells leads to reduced levels of calveolin-1, a protein that controls EGFR inactivation via receptor internalization [90]. This downregulation of calveolin-1 impairs EGFR internalization enabling EGFR activation [90]. Bartolomé et al. have recently reported that α2β1 can interact with cadherin-17 (CDH17) and that this binding leads to an increase in cyclin D1 and proliferation in colon cancer cells that metastasize to the liver [91].

The most well described cytokine involved in the expression of pro-proliferating integrins in cancer is tumor necrosis factor-α (TNF-α), which is a pro-inflammatory cytokine that is involved in tumor associated inflammation [92]. TNF-α induces the expression of the β1 integrin subunit and specially, α2β1 integrin via the MAP kinase pathway (Figure 3(2)) [93]. It is broadly accepted that β1 integrin plays an important role in promoting proliferation and transformation. For instance, β1 expression regulates type 1 insulin-like growth factor receptor (IGF-IR) via Grb2-associated binder-1 (Gab1), which is responsible for the inhibition of phosphorylation of IGF-IR, leading to anchorage-independent growth of prostate cancer [94]. Further on, downregulation of β1 inhibits IGF-IR and AKT activation [95].

Although alterations in integrin expression are found in different types of cancer, there is not much evidence about exogenous compounds that induce alterations in integrin expression and that affect directly cell proliferation. The most prominent example might be the cell treatment with the plant- derived compound phorbol ester. Upon treatment with this substance, promonocytic cell lines induced cellular adherence and growth inhibition via upregulation of the β2 integrin subunit [96]. Fish oil fatty acids (e.g., ω3 and ω6 polyunsaturated fatty acids) were shown to inhibit in vitro integrin-mediated proliferation by repressing ILK in human lung cancer [97]. Of note, regarding endogenous components that modulate integrin-mediated proliferation, it was found that cell treatment with calcitriol (vitamin D) inhibited cell proliferation and downregulated α1 integrin subunit in colon cancer cells and cultured hepatic stellate cells [98,99]. In prostate cancer cells, treatment with calcitriol downregulated α6β4 via downregulation of parathyroid hormone-related protein (PTHrP) [100]. These components should be further explored and might represent interesting supplements for cancer therapies.

### 2.2. Evading Growth Suppressors

In order to be able to tightly control tissue architecture and function, cells do not only need to receive signals to start proliferation and progress through the cell cycle, but also to respond to anti-growth signals such as contact inhibition or activation of tumor suppressors [5].

Almost a half century ago, it was already known that cells stop proliferation and movement if they get in touch with other cells [102]. The extracellular proteins nectin-3 and nectin-like molecule-5 (Necl-5) are involved in a phenomenon known as contact inhibition of locomotion. Necl-5 interacts heterophilically with nectin-3 when proliferating cells collide to another [103]. Necl-5 is then downregulated and nectin-3 *trans*-interacts with nectin-1, leading to the inactivation of αvβ3 integrin, recruitment of cadherins and formation of adherent junctions [103]. In cancer, Necl-5 can interact *in cis* with αvβ3 integrin and induce clustering at the leading edge of a moving cell, thus enhancing migration [104]. Additionally, Necl-5, integrin αvβ3 and platelet-derived growth factor receptors (PDGFR) formed a complex that inhibits RhoA in an Src-dependent manner upon platelet-derived growth factor (PDGF) stimulation, allowing cells to migrate [105]. Dysregulation of contact inhibition of locomotion was found in vitro in prostate cancer cells via activation of EphB3 and EphB4 and in vivo, this was observed in embryonic fibroblasts and neural crest [106,107].

Cells also exhibit another mechanism of control known as contact inhibition of proliferation. The tumor suppressor Merlin, encoded by the gene neurofibromatosis type 2 (NF2) is responsible for the regulation of EGFR in response to cell contact [108]. The mechanism of this occurrence is not yet understood but it was observed that Merlin binds to EGFR, blocking its internalization and this might immobilize the EGFR on the plasma membrane and stabilize cell junctions [108]. NF2 mutations are found in different pathologies, especially in malignant mesothelioma and Neurofibromatosis type 2. In Neurofibromatosis type 2, Merlin was found to co-localize with β1 integrin [109]. However, recent in vitro experiments showed that integrin-mediated adhesion to fibronectin was sufficient to inactivate Merlin and activate mTORC1 signaling pathway [110]. Further on, Merlin might also regulate integrin activity by binding to paxilin bound to β1 and HER1 at the plasma membrane [111]. Merlin was also shown to interact with another protein known as Kibra and to activate the Hippo pathway, which is involved in cell contact inhibition and tissue growth control [112]. The final goal of this signaling pathway is to achieve the translocation of a transcription factor known as YAP into the cytoplasm, so that it cannot bind to the DNA and promote transcription of growth promoting genes [113,114]. Additionally, it was found that integrin α6β1-mediated binding to the extracellular matrix component laminin-511 produced by breast cancer stem cells activates the Hippo transducer TAZ [115]. Further on, it was found that integrin α5 subunit is a target gene of TAZ. TAZ promotes formation of laminin-511 [115]. In this case, expression and ligand binding of integrin α6β1 might be a crucial step to initiate an uncontrolled and self-sustaining mechanism of growth of breast cancer stem cells.

The most commonly mutated tumor suppressor gene in cancer is *TP53*. Not only do wildtype and mutated p53 lose the ability to inhibit cell cycle progression but also they can acquire the ability to promote tumorigenesis [116]. Several groups have reported that there is a crosstalk between integrins and p53 that determines cell fate. In epithelial cells, apoptosis is induced if cells detach from the ECM (“ligand-free integrins”) or if DNA damage occurs when cells are attached to the ECM via integrins and wildtype p53 is present in the cell [117]. However, in some melanoma and sarcoma cell lines, wildtype p53 levels decrease upon cell detachment, leading to less sensitivity to DNA-damage and promoting cell survival [117]. Supporting these observations, Bachelder et al. demonstrated that ectopic expression of integrin α6β4 in colon and breast cancer cell lines triggers wildtype p53-mediated apoptosis, suggesting that ligand-free α6β4 might activate p53 [118]. Moreover, mutations in the TP53 gene also lead to an altered interaction with integrins. For instance, Muller et al. have showed that mutant p53 leads to an increased recycling of α5β1 and EGFR by indirectly promoting the interaction between α5β1 and the Rab11 effector Rab-coupling protein (RCP), which is necessary for the EGFR-α5β1 recycling to the plasma membrane [119]. This faster recycling leads to an increased cancer cell invasion and random migration [119]. In glioblastoma multiforme, another interplay between α5β1 and p53 was observed [120]. Tumors with wildtype p53 and high expression of α5 integrin are resistant to temozolomide chemotherapy [120]. However, inhibition of the mouse double minute homolog (MDM)-p53 complex by the small inhibitor Nutlin-3 restores sensitivity to temozolomide chemotherapy and reduces α5 expression [120]. In ovarian cancer, mutated p53 enables α5β1 integrin-mediated anchorage independent growth [121].

### 2.3. Activating Invasion and Metastasis

Unlike mesenchymal cells, epithelial cells have the ability to migrate only during development or tissue renewal, such in the gut or wound repair [122]. To be able to migrate, tumor cells have to acquire the ability to get rid of the cell-cell contacts, cross the basal membrane, cross the stroma, enter the circulatory system, invade a new distal site and colonize the new organ. Many excellent reviews on these issues have recently been published [123,124,125,126,127] and thus, we will present here some mechanisms which lead to integrin expression and which might initiate tumor invasion.

The first step involved in cell invasion is known as epithelial-mesenchymal transition (EMT) [128]. One of the major EMT inducers is the cytokine TGF-β1 [128]. TGF-β1 resides in the ECM in an inactive form bound to two peptides known as latency-associated peptide (LAP) and one of four latent TGFβ-binding protein (LTBP) [126]. Integrins αvβ3, αvβ5, αvβ6, αvβ8 and an unidentified β1 can bind to the tripeptide Arg-Gly-Asp (RGD)-motif of the LAP protein, inducing a conformational change and exposing TGF-β1 to the adjacent cells [126,129,130]. Upon binding to the receptor, TGF-β induces downregulation of epithelial proteins such as E-cadherin and upregulation of mesenchymal proteins such as N-cadherin. TGF-β1 upregulation leads not only to an upregulation of several integrins, such as the αv and β6 integrin subunits [131], but also to an alteration of components of the ECM, which in turn are integrin ligands [132]. In vitro, TGF-β1 was found to increased levels of αvβ3 integrin, PI3K, Akt and NF-kappaB-dependent pathway [133]. Further on, TGF-β signaling upregulates EGFR, which enhances malignancy, as it was demonstrated that elevated EGFR levels were sufficient to transform breast cells in vitro [134]. Moreover, during EMT and the reverse mechanism known as mesenchymal-epithelial transition (MET), FGFR1 was found to be upregulated upon stimulation with TGF-β1 in a breast cancer cell model [135]. A novel study showed that FGFR is associated to E-cadherin, but under expression of β3 integrin subunit, which is upregulated upon TGF-β stimulation [136], FGFR dissociates from E-cadherin and associates with β3 integrin, leading to Erk1/2 phosphorylation in response to FGF2 [137,138]. Bone morphogenic protein 7 (BMP-7) is a cytokine that belongs to the TGF-β superfamily [139] and was shown to induce invasion in hepatocellular carcinoma cells [140], breast cancer cells [141] and chondrosarcoma cells [142]. In chondrosarcoma cells, BMP-7 enhances αvβ3 integrin expression through the c-Src/PI3K/Akt/IKK/NF-κB signaling pathway [142]. In breast cancer cells however, αvβ3 expression depends on TGF-β2 induction and its expression was shown to be sufficient and necessary to activate Slug, a transcription factor that induces EMT [143].

Cantor et al. analyzed the proteome of colorectal cancer cell lines after overexpression of the β6 integrin subunit [144]. They found that this overexpression was sufficient to enhance proliferation and decrease cell adhesion to the ECM [144]. Of note, TGF-βR1 was upregulated (and not TGF-β1) [144]. Additionally, the downregulation of IGF2R and upregulation of glutathione-S-transferase pi 1 might contribute to the proliferative phenotype [144].

Recently, a novel mechanism for regulation of EMT-MET was reported. RAD21 is a subunit of the cohesion complex which is responsible for maintaining the correct structure of chromatids during the S-phase, mitosis and meiosis [131]. Yun et al. reported that RAD21 is expressed in epithelial breast cancer cells but not in mesenchymal cancer cells and showed that depletion of RAD21 in epithelial cancer cells created a permissive transcriptional environment within the *TGFB1* and *ITGA5* loci, leading to a higher expression of these integrins [145]. Shibue et al. demonstrated that metastatic cells in the lung proliferation are mediated by β1-mediated signaling [146]. After successfully achieving a mesenchymal phenotype, tumor cells can initiate migration. In this context, the microenvironment plays an essential role. Carcinoma associated fibroblasts (CAFs) contributes to migration by secreting growth factors (such as HGF and PDGF) that induce survival, proliferation and motility in tumor cells, by inducing angiogenesis via VEGF and altering the ECM [147]. Recently, it was shown that CAFs led migration of colon cancer cells via surface associated FGF-2, which binds to the FGFR located on the surface of cancer cells, activating SRC and inducing integrin αvβ5 expression which leads to adhesion of colon cancer cells to fibroblasts [147].

Tumor cells per se are also able to modify the ECM so that they can migrate through it. Ovarian cancer cells with elevated levels of αvβ6 showed in vitro a higher expression of urokinase-type plasminogen activator (uPA), uPA-receptor (uPAR) and matrix metalloproteinases (MMP) such as MMP-2 and MMP-9 [148]. Upregulation of MMP-2 and MMP-9 caused by overexpression of αvβ6 was also observed in squamous carcinoma cells [149]. Recently, Dutta et al. showed that αvβ6 is necessary to express TGFβ1-mediated MMP-2 by binding to TGFβRII and activating Smad3 [150]. In line with these results, it was shown in an in vivo prostate cancer model that αvβ6 induces MMP2 which contributes to osteolysis in prostate cancer bone metastasis [151].

Not only does uPA-uPAR cascade play a crucial role in ECM degradation and tumor invasion but also in angiogenesis, inflammation, immunity and coagulation [152,153,154,155]. In tumor cells, uPAR modulates β1 and β3 integrin signaling by binding to these integrins in a vitronectin-dependant manner [156]. uPA-mediated ECM degradation confers the invasive cells a mesenchymal phenotype. However, a recent study reported that uPAR-integrin interactions are necessary to confer malignant cells with the so-called *amoeboid invasion*, which enables malignant cells to move fast through the tissue [157]. Furthermore, uPAR interacts with the α5β1-fibronectin complex, leading to a constitutive ERK1/2 activation [158]. Downregulation of uPAR was shown to be sufficient to induce a cancer cell dormant state [158].

### 2.4. Limitless Replicative Potential

Another characteristic of cancer cells is that they can proliferate indefinitely, while normal cells have limited proliferation capacity [159]. This phenomenon has already been observed by Hayflick et al. in 1961 [159] and to date, it is common accepted that cells can enter senescence or crisis [5,6]. This brake in proliferation can be induced upon DNA damage or aging [160]. Further on, normal cells have an internal biological clock in form of repetitive DNA sequences at the end of chromosomes which are known as telomeres [161]. The telomeres act as protecting cap and because the replication machinery of the cell, can not copy this ends, they get shorter with each cell division [162]. When telomeres achieve a critical length, cells recognize them as DNA damage, activate the tumor suppressor p53 and cells enter senescence or apoptosis [160]. Telomere sequences can be prolonged via a reverse transcriptase known as telomerase [163]. It is generally accepted that only few normal cell types such as male germ-line spermatocytes and around 90% of all cancers exhibit telomerase activity [164,165]. In adult mice, telomerase was detected in a positive α6-integrin subpopulation containing spermatogonia and enriched in spermatogonial stem cells [166].

Regarding the role of integrins in the limitless replicative potential of cancer cells, Ponnala et al. made a significant contribution. This group showed that downregulation of MMP-9 in glioblastoma cells leads to a downregulation of hTERT and that this is mediated by β1 integrin [167]. What is more, downregulation of hTERT via siRNA in cancer cell lines leads to the inhibition of cell growth and proliferation [168]. In this study, silencing of hTERT lead to the downregulation of the integrin αV, among other genes [168].

### 2.5. Sustained Angiogenesis

Cancer cells as well as normal cells need a constant supply of nutrients and oxygen to be able to live. It is widely accepted that tumors lack the ability to exceed a diameter of 2 mm in the absence of functional blood vessels. They overcome this limitation by inducing the formation of new vessels from pre-existing ones [160]. The formation of new vessels facilitates tumor survival, tumor invasion, and metastasis. Endothelial cells that are in the surroundings of the tumor are in a quiescent state. However, when tumor cells start secreting pro-angiogenic molecules, there is an imbalance of pro-angiogenic and anti-angiogenic molecules which leads to an angiogenic switch [169,170]. VEGF and FGF2 and their receptors are the most potent activators of angiogenesis and these signaling axes promote several mechanisms that contribute to the formation of new vessels, such as the remodeling of the ECM via MMPs and uPA and migration and proliferation of endothelial cells [171]. In this context, three endothelial integrins play crucial roles: αvβ3, αvβ5 and α5β1 [172,173]. VEGF-mediated angiogenesis occurs via αvβ5, while FGF-mediated angiogenesis occurs via αvβ3 and α5β1 [174]. The integrin-mediated processes involved in tumor angiogenesis are reviewed somewhere else [172,173,175]. Most of the articles related to tumor angiogenesis focused on the integrins being upregulated on the endothelium, which promote migration and proliferation of endothelial cells. The role of integrins on endothelial cells and/or promising therapies targeting these pro-angiogenic integrins have already been discussed by us as well as by other authors in several publications [161,162,163,164,165,176]. For this reason, in this review, we will further analyze the opposite side of the coin and will only pinpoint which integrins have their expression altered in tumor cells during processes that trigger tumor angiogenesis.

In cancer, hypoxia might be the major initiator of tumor angiogenesis. When cells are exposed to a low oxygen partial pressure, the hypoxia-inducible factors (HIF-1α, HIF-2α, and HIF-3α) are stabilized and translocate into the nucleus [177,178]. Once in the nucleus, these factors can bind to hypoxia response elements (HREs) and regulate at least transcription of 70 different genes [179]. On one side, this leads to an upregulation of specific integrins such as αvβ3 and the β1 and α6 integrin subunits, which mediate the invasive phenotype of tumor cells [180,181,182]. Moreover, a recent study revealed that HIF-1α activates ILK transcription and that the activation of this kinase stimulates HIF-1α expression via the mTOR pathway, creating a sustained feedback loop that promotes EMT [183]. Another feedback loop was reported in glioblastoma cells [184]. Under hypoxic conditions, αvβ3 or αvβ5 integrins are recruited to the plasma membrane and regulate HIF-1α cellular levels through inhibition of GSK3-β mediated by a small GTPase known as RhoB, which is activated by FAK [184].

Regarding the activation of tumor angiogenesis, the most prominent targets of HIF-1α are vascular endothelial growth factor (VEGF), calcitonin receptor-like receptor (CRLR), stem cell factor (SCF) and angiopoietin 2 (ANGPT2) [185,186,187,188]. Of note, VEGF does not only exert its effect on endothelial cells but also on tumor cells. There is an autocrine and paracrine signaling loop that promotes tumorigenicity (reviewed in [189]). In this context, specific integrins such as α6β1, αvβ3 and α9β1 cooperate with growth receptors and activate several integrin-mediated pathways [189,190].

### 2.6. Evading Apoptosis

Under normal conditions, non-activated epithelial cells need a substrate to attach to ensure cell polarity and cell survival. Per default, the apoptotic cell program must be suppressed and survival signals are required to ensure cell viability [191]. Herein, we will focus on anoikis, which is the process by which cells undergo a controlled death upon loosing contact with the ECM. Integrins are the key protagonists of this process. When cells adhere to the ECM via integrins, the FAK-PI3K-AKT and FAK-MAP kinase pathway are activated, preventing cells from death [192]. Benoit et al. clearly explained this phenomenon in a recent review [193]. Integrin α8β1 is expressed in intestinal crypt epithelial cells and upon ligand binding, it recruits vinculin to the focal adhesion complex forming a complex with paxilin [193,194]. FAK is activated and turns on the PI3K/AKT pathway, leading to cell survival. If there is no ligand, α8β1 has a different conformation and FAK can not be activated and the PI3K-AKT pathway is switched off [193,194]. Further on, if there is no ligand, the integrin subunits β1and β3 expressed in adherent cells recruit a protein involved in apoptosis known as caspase-8 to the plasma membrane and activate it, leading to integrin-mediated cell death [195]. In keratinocytes, caspase-8/β1 interaction leads to an internalization and progressive degradation of β1, leading to cell death [196].

**Figure 4:**
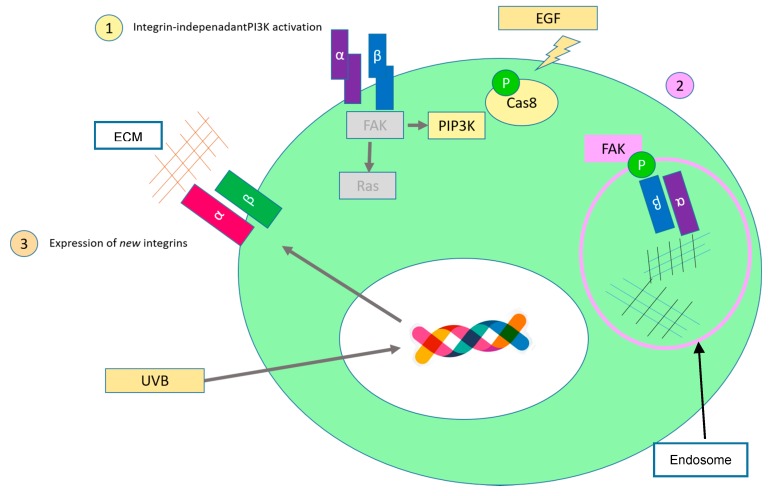
Schematic representation of some integrin-mediated mechanisms to avoid anoikis. (1) Growth factors can phosphorylate caspase-8 and this allows interaction and activation with PI3K; (2) Integrins are endocytosed and integrin signaling continues in the endosomes via interaction with FAK; (3) Integrins that are not expressed in a specific tissue are upregulated. These integrins can bind to new components of the extracellular matrix (ECM) and start signaling. (Of note, the DNA cartoon was designed by Freepik). Big ellipse in green: cell; small ellipse in white: nucleus.

Cancer cells develop different strategies to overcome controlled cell death, such as upregulation of receptor tyrosine kinases or small GTPases [197]. Cancer cells that detach from the substrate can avoid anoikis by downregulating caspase 8 via promoter methylation [198]. This feature was found in childhood neuroblastomas with amplification of the oncogene MYCN [198]. However, mutations are very rare and other studies revealed that caspase-8 has a non-canonical function by promoting cell migration [199,200]. Another mechanism exhibited by cancer cells is the phosphorylation of caspase-8 induced by EGF that enables interaction with PI3K, promoting cell migration (Figure 4(1)) [200]. A novel study revealed recently that cells can overcome anoikis via endocytosed active integrins that continue signaling in endosomes [201] (Figure 4(2)). In this work, Alanko et al. demonstrated that phosphorylated FAK localizes to Rab21-dependant endosomes containing active β1-integrin bound to its ligand [201,202]. Another mechanism to evade cell death is the alteration of the integrin repertoire, such as overexpression of the β1 integrin subunit [203]. Early studies showed that oncogenic signaling leads to changes in the integrin heterodimers, as we described before in the case c-myc-mediated expression of α1β1 integrin in colon cancer [204,205]. External stimuli can also alter the integrin expression patterns. For instance, human melanocytes express αvβ3 at low levels and can not attach to the collagen-rich dermis. This promotes apoptosis. However, melanoma cells upregulate αvβ3 expression after ultraviolet B(UVB) exposure enhancing melanoma cell adhesion and migration (Figure 4(3)) [206]. In brain capillaries, hypoxia leads to overexpression of α5β1 and mechanical stress leads to upregulation of β1 in gliomas and breast cancer cells [207,208].

## 3. Hotspots for Anticancer Treatment

In this review, we discuss some advanced mechanisms that envision how integrins contribute to all the Hallmarks of Cancer once proposed by Hanahan and Weinberg [5] (Table 2). In cancer, integrins are deregulated in part by enhanced transcription induced by oncogenes, alterations in the chromatin structure, overexpression of growth factors and growth factor receptors or changes in the ECM. The indispensable role of integrins in carcinogenesis is also exposed by the fact that integrin single-nucleotide polymorphisms (SNP) seem to have a great impact in tumor agressiveness, survival and suceptibility to thrombus formation in cancer [209,210,211,212]. It is, therefore, unquestionable that integrins are promising targets for cancer treatment.

Several efforts have been made to develop integrin antagonists so that one or all hallmarks of cancer are inhibited. Table 3 summarizes the results from the clinical trials performed so far using antibodies targeting integrins in cancer. Of note, we found a patented chitosan polymer covalently linked with small molecule integrin αvβ3 and αvβ4 antagonist for targeted delivery of drugs, nucleic acids or other compounds to cells expressing these integrins (patent: EP2806896) [213]. However, we were not aware if this compound is currently being clinical evaluated for cancer treatment. The most prominent compound targeting integrins might be cilengitide, a cyclic RGD peptide that targets integrins αvβ3 and αvβ5 [214]. We and other groups could show in vitro and in vivo that this peptide inhibits attachment and invasion of different tumor cells [215,216,217,218,219,220,221]. However, cilengitide failed to provide benefit in a clinical trial phase III (CENTRIC study) [222]. The work of Reynolds et al. presented a novel mechanism by which integrin are regulated that might explain at some extent the failure of cilengitide in the late-stage clinical trial CENTRIC [223]. This group elucidated that low concentrations of cilengitide alter αvβ3 integrin and VEGFR-2 trafficking, leading to enhanced angiogenesis [223]. This discovery highlights the crucial role of integrin trafficking (reviewed in [224]) and might explain why targeting integrins in cancer has not met the clinical expectations yet. In accordance to the vital role that integrins fulfill in the organisms, it seems evident that cells have developed strategies to compensate integrin inhibition to ensure cell survival, as we discussed above regarding the interplay between α1β1/calveolin-1/EGFR [90]. Although α1β1 could be *prima facie* an interesting target in cancer, the aforementioned evidence shows that targeting this integrin could have the opposite effect.

Besides antibodies and peptides antagonizing integrins, other integrin-targeted strategies, such as oncolytic virus, are starting to emerge. Recently, Tian et al. presented a tobacco mosaic virus conjugated with a cyclic RGD and the chemotherapeutic agent doxorubicin [232]. The virus entry is mediated by integrins through binding to the RGD motif and this shows antitumor efficiency in vitro and in vivo [232]. The adenovirus DNX-2401 (former name: Δ-24-RGD-4C) is an oncolytic virus that is competent in cells with defects in the tumor suppressors Rb and p16 and uses an RGD-motif that mediates entry via integrins into the tumor cells [233,234]. At the moment, there are two clinical trials testing its efficacy. The TARGET-I study (NCT02197169) is a phase Ib study to evaluate the combination treatment of DNX-2401 (DNAtrix) with interferon gamma (IFN-γ) for recurrent glioblastoma and gliosarcoma and the CAPTIVE study (NCT02798406) is a phase II trial which evaluate the effects of the combination of DNX-2401 and Pembrolizumab in gliosarcomas and glioblastomas. Another oncolytic virus that is now being investigated in clinical trials is CAVATAK (Viralytics, Sydney, Australia). CAVATAK targets cells with high levels of ICAM-1 and its anti-tumor effects resides in triggering an anticancer immune response. CAVATAK efficacy is being evalatued in clinical trials to treat stage IIIc and IV malignant melanoma (NCT01227551), to treat non-small cell lung cancer and bladder cancer in combination with Pembrolizumab (STORM/KEYNOTE-200) (NCT02043665) and to treat advanced melanoma in combination with ipilimumab (NCT02307149).

## 4. Concluding Remarks

The evidence presented in this review clearly shows that integrins are hot targets for anticancer therapies. However, the crucial role of integrins seems to be supported by a plethora of mechanisms that counteracts integrin inhibition. Currently, it might be evident that targeting one or more integrins alone will not have curative effects, but it will do it in combination with other agents. Furthermore, the therapeutic strategies discussed above with oncolytic virus using integrins as “cell openers” of tumor cells might circumvent the complexity of integrin-mediated signaling and promise a hopeful future regarding cancer treatment.

## Figures and Tables

**Table 1:** Crosstalk between growth factor receptors and integrins.

Type of Interaction	Interaction Partner	Integrin	Cell Line	Description and References
Direct interaction between integrins and growth factor receptors and potentiation of signaling pathways	IGF-1R	α6β4	MCF-7	Integrin-IGF1-IGF1 receptor ternary complex formation [63].
	FGFR	αvβ3	K562	FGFR1-FGF1-integrin αvβ3 ternary complex formation [64].
	c-Met	α5β1	SKOV3ip1 and HeyA8	Upon binding to fibronectin, α5β1-integrin interacts directly with the receptor tyrosine kinase c-Met and activates it in a ligand-independent manner [65]. (Figure 3(1))
Regulation of expression level of growth factor receptors by integrins	EGFR	β1	A549	Downregulation of the β1 subunit leads to an increased level of the EGFR at the plasma membrane [66].
	EGFR	α6β4	AsPC1, Suit-2, and Panc-1	Integrin α6β4 leads to the recruitment of the c-Cbl ubiquitin ligase to the growth factor receptor and this leads to a reduced EGFR degradation [67].
	EGFR	α6β4	MDA-MB-231	Crosslinking of integrin induces EGFR clustering and promotes EGF-mediated signaling [68].
Activation of integrins via growth factor receptor signalling	EGFR	αvβ5	FG	EGFR ligand binding induces Rap1 activation and this leads to the activation of αvβ5 [69].

IGF-1R: insulin-like growth factor 1; FGFR: fibroblast growth factor receptor; c-Met: met proto-oncogene/hepatocyte growth factor receptor; EGFR: epidermal growth factor receptor; MCF-7: breast cancer cell line Michigan Cancer Foundation-7; MDA-MB-231: breast cancer cell line; FG: pancreatic carcinoma cell line.

**Table 2:** Summary of the contributions of integrins in the Hallmark of Cancer. (The reader will find all the references in the main text).

Hallmark of Cancer	Integrin’s Contribution
Sustaining proliferative signalling	Integrins can potentiate growth factor signalling pathways that promote proliferation via (1) direct binding and synergy; (2) modulation of expression levels of growth factor receptors or (3) by direct activation of growth factor receptors. The oncogene MYC and cytokines such as TNF-α can upregulate pro-proliferating integrins. Integrin interactions with other proteins such as CD98hc and transmembrane CDH17 that promote proliferation. Exogenous and endogenous compounds (e.g., phorbol ester, fish oil fatty acids and vitamin D) can alter integrin expression and modulate cell proliferation.
Evading growth repressors	Dysregulation of contact inhibition of locomotion: Necl-5 interacts with αvβ3 (and not with nectin-3 in trans), promoting cell migration. Dysregulation of contact inhibition of proliferation: Engagement of integrins in fibronectin binding is sufficient to inactivate Merlin, the protein responsible for stabilizing cell junctions. In turn, Merlin might also modulate β1 integrin located at the plasma membrane. Crosstalk with p53: - Integrin-mediated ECM detachment can lead to a decreased level of wildtype p53. This promotes cell survival beyond DNA-damage. - Restoration of normal wildtype p53 levels decreases α5 levels and sensitizes glioblastoma cancer cells to a specific chemotherapeutic agent. - Mutated p53 alters integrin recycling and promotes cancer cell migration and invasion.
Invasion and metastasis	Role of integrins during EMT: - Specific integrins can initiate a paracrine loop of TGF-β1, one of the major EMT inducers. In turns, TGF-β1 induces an upregulation of specific integrins that promote a malignant phenotype. - Some integrins subunits can upregulate TGF-βR1 and Slug, both inducers of the EMT. Role of integrins during migration/invasion: - FGF-2 bound to CAFs activates the FGFR signalling pathway in colon cancer cells and leads to an upregulation of integrins that can adhere to fibroblasts and migrate. - αvβ6 enhances expression of uPAR and MMP-2 and MMP-9 that are responsible for ECM degradation, enabling migration as a result. - uPAR-integrin interaction is necessary to acquire an invasive amoeboid phenotype.
Limitless replicative potential	Downregulation of hTERT leads to downregulation of the αv integrin subunit. MMP-9 decreases β1 integrin and the subsequent FAK activation decreases hTERT expression.
Sustained angiogenesis	Hypoxia initiates a signaling cascade that upregulates specific integrins and pro-angiogenic factors such as VEGF and ANGPT2. The upregulated integrins mediate tumor cell invasion and regulate also HIF-1α cellular levels. VEGF is also involved in an autocrine and paracrine loop which is mediated by integrins. The secreted compounds by the tumor cells induce an angiogenic switch and upregulate integrins which are involved in endothelial cell migration and proliferation.
Evasion of apoptosis	Promoter methylation of caspase-8. Phosphorylated caspase-8 (induced by EGF) leads to PI3K activation. Expression of new integrins so that cells can bind to other ligands via integrins.

**Table 3:** Results of clinical trials targeting integrins in cancer.

Compound	Target	Stage	Result	Reference
Vitaxin (Etaracizumab)	αvβ3	Phase II	No clinically meaningful improvement in survival.	[225]
CTNO 95 (Intetumumab)	αv (αvβ3 and αvβ5)	Phase II	No clinically meaningful improvement in survival.	[226]
Volociximab	α5β1	Phase II	Insufficient clinical activity.	[227]
ATN-161	α5β1	Phase I	1/3 of patients manifested stable disease.	[228]
			Two Phase II trials were discontinued in February 2016.	
PF-04605412	α5β1	Phase I	Trial was prematurely terminated.	[229]
IMGN388	αv integrin-targeting antibody conjugated to a cytotoxic agent	Phase I	Well tolerated but it was discontinued.	[230]
GLP0187	Several αv integrins	Phase I	No signs of monotherapy efficacy.	[231]

## Abbreviations

ECM	Extracellular matrix
FAK	Focal adhesion kinase
Rac1	Ras-related C3 botulinum toxin substrate 1
MAPK	MAP kinase pathway
EGF	Epidermal growth factor
PI3K	Phosphatidylinositol 3-kinase
PDK1	Phosphoinositide-dependent kinase-1
mTOR	Mammalian target of rapamycin
VEGF	Vascular endothelial growth factor
YAP	Yes-associated protein
PIP3	Phosphatidylinositol-3-phosphate
GSK3	Glycogen synthase kinase 3
PHI-1	Phosphatase holoenzyme inhibitor 1
PIP2	Phosphatidylinositol 4,5-bisphosphate
CDH17	Cadherin-17
TNF-α	Tumor necrosis factor-α
IGF-IR	Insulin-like growth factor receptor
Gab1	Grb2-associated binder-1
HGF/SF	Hepatocyte growth factor/scatter factor
Necl-5	Nectin-like molecule-5
NF2	Neurofibromatosis type 2
PAK	p21-activated kinase
EMT	Epithelial-mesenchymal transition
LAP	Latency-associated peptide
LTBP	TGFβ-binding protein
BMP-7	Bone morphogenic protein 7
CAF	Carcinoma associated fibroblasts
HREs	Hypoxia response elements
CRLR	Calcitonin receptor-like receptor
SCF	Stem cell factor
ANGPT2	Angiopoietin 2
uPA	Urokinase plasminogen activator
uPAR	Urokinase plasminogen activator receptor
MMP	Matrix metalloproteinases
SNP	Single-nucleotide polymorphisms
